# Age-defying swallowing

**DOI:** 10.3389/fragi.2025.1510257

**Published:** 2025-04-07

**Authors:** María-Itatí Palacio, Rosa-María Bermejo, Ana-María Lucas-Ochoa, Ana-María González-Cuello, Emiliano Fernández-Villalba, María-Trinidad Herrero

**Affiliations:** Clinical and Experimental Neuroscience Group (NiCE), Department of Human Anatomy and Psychobiology, Biomedical Research Institute of Murcia (IMIB), Institute for Aging Research, School of Medicine, University of Murcia, Murcia, Spain

**Keywords:** presbyphagia, ageing, swallowing, prevention, new technologies, dysphagia

## Abstract

Swallowing disorders, which are generally underdiagnosed, affect the elderly, leading to a decreased quality of life and complications, including aspiration pneumonia and death. Understanding the neurophysiology of swallowing and the causes of its dysfunction is a fundamental tool for the prevention, early diagnosis, and treatment of dysphagia. New technologies open a wide range of possibilities for the implementation of new care protocols for this disorder.

## 1 Introduction

All around the world religious, cultural and familial traditions play a significant role in eating, drinking and swallowing (Jardine et al., 2020). The ease with which we and swallow is surprising given the complex neurological control system involved. The development of feeding and swallowing involves a set of interactions that begin in the embryonic and fetal periods and continue throughout childhood until mature swallowing is achieved (Reid et al., 2016).

Oropharyngeal dysphagia is a swallowing disorder defined by the difficulty in safely and efficiently forming and/or moving the food bolus from the oral cavity to the esophagus. Presbyphagia, on the other hand, is the swallowing disorder caused by normal ageing. In patients with dysphagia or presbyphagia, quality of life, eating habits and diet are altered. The health of these patients deteriorates and, in many cases, these disorders lead to aspiration pneumonia and death (Clavé et al., 2015).

The economic consequences of dysphagia in hospitalized patients are significant, as patients with dysphagia generate costs up to 48% higher than those without dysphagia and are 33.2% more likely to be transferred to a post-acute care facility. Dysphagia increases the likelihood of a longer hospital stay and even death in patients who suffer from it (Patel et al., 2018). In their daily lives, people affected by undiagnosed dysphagia or presbyphagia may make intuitive adjustments to their diet, use strategies to control food intake and be able to eat small amounts of food and liquids (Ballesteros-Pomar et al., 2020). However, in the context of additional deterioration, particularly an acute health change or surgery, swallowing problems can destabilize and have a negative impact on overall diagnosis, prolong hospital days, increase hospital readmissions, increase the need for prolonged treatments and require long-term rehabilitation care (Allen et al., 2020).

Therefore, understanding the anatomical and physiological processes of swallowing and its alterations is essential, especially in older adults (Matsuo and Palmer, 2008).

## 2 Neuroanatomy and neurophysiology of swallowing

Swallowing is the safe transport of food and liquids from the oral cavity to the esophagus (Ambrocio et al., 2023). It is not only essential for nutritional intake but also plays a role in managing internal secretions of the upper (saliva, bile, and nasal secretions) and lower (tracheal or bronchial) aerodigestive tracts. The physiological mechanism of swallowing is divided into four well-defined phases: the oral preparatory phase, the oral transport phase, the pharyngeal phase, and the esophageal phase; the first two are voluntary, and the last two are reflexive (Costa, 2018). For the motor program of swallowing to be adequate and aligned with the needs of each moment, the coupled participation of various cortical and cranial areas, five cranial nerves (V, VII, IX, X, and XII), the first three cervical segments, and the muscles of the mouth, pharynx, and esophagus is necessary, coordinated among themselves from the nuclei of the brainstem (Tables 1, 2) (Krishnamurthy et al., 2021).

**TABLE 1:** Schematic representation of the muscles involved in each phase of swallowing and their motor innervation. Muscles 7 (a, b and c), 8 (a, b and c), 13, 15–17, and 20 are not depicted in the diagrams. Colors correspond to the innervation of each cranial nerve*.*

Phase	Action	Muscle	Motor function	C. N
Oral (Preparatory and Transport) 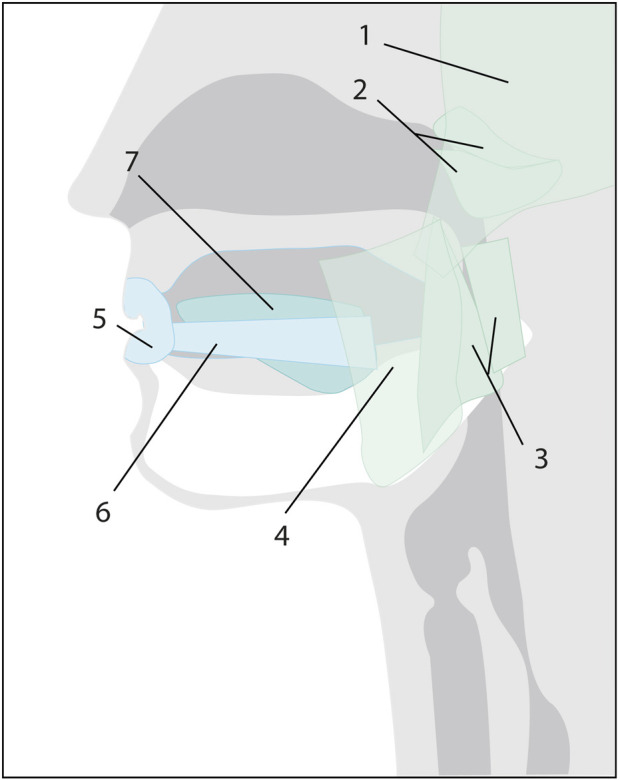	Chew food (jaw)	1. Temporalis	Jaw closure	V
		2. Medial Pterygoid	Jaw closure	V
		3. Lateral Pterygoid	Jaw opening, protrusion	V
		4. Masseter	Jaw closure, protrusion	V
	Close mouth	5. Orbicularis oris	Purses lips	VII
		6. Buccinator	Flattens cheeks	VII
	Move food With tongue	7. Intrinsic of the tongue: 7a.longitudinal, 7b.vertical, 7c.transverse	Alters the shape of the tongue	XII
Pharyngeal 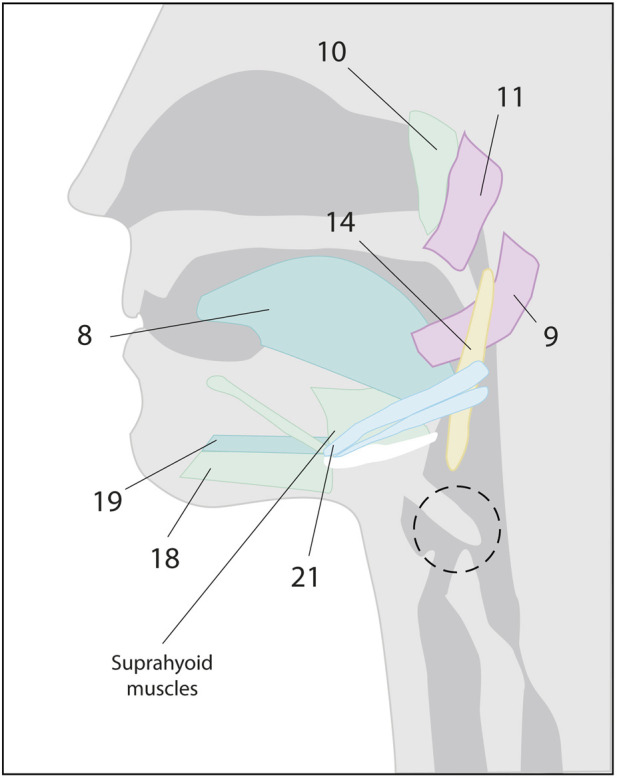	Push food into Pharynx (tongue)	8. Extrinsic of the tongue: 8a.genioglossus, 8b.hyoglossus, 8c.styloglossus	Alters the position of the tongue in the mouth	XII
		9. Palatoglossus	Elevates posterior tongue during swallowing	X
	Close nasopharynx (palate)	10. Tensor veli palatine	Stiffens soft palate	V
		11.Levator veli palatine	Elevates soft palate	X
		12. Palatopharyngeus		X
		13. Musculus uvulae	Closes nasopharynx	X
	Open pharynx	14. Stylopharyngeus	Elevates larynx and pharynx during swallowing	IX
		15. Salpingopharyngeus	Shortens and widens pharynx	X
		16. Palatopharyngeus	Shortens and widens pharynx	X
	Elevate hyoid	17. Digastric (anterior belly)	Elevates hyoid bone	V
		18. Mylohyoid	Elevates hyoid, tongue	V
		19. Geniohyoid	Elevates hyoid bone forward, helps depress mandible	XII
		20. Digastric (posterior belly)	Elevates hyoid, depresses mandible	VII
		21. Stylohyoid	Elevates hyoid and tongue	VII
Esophageal 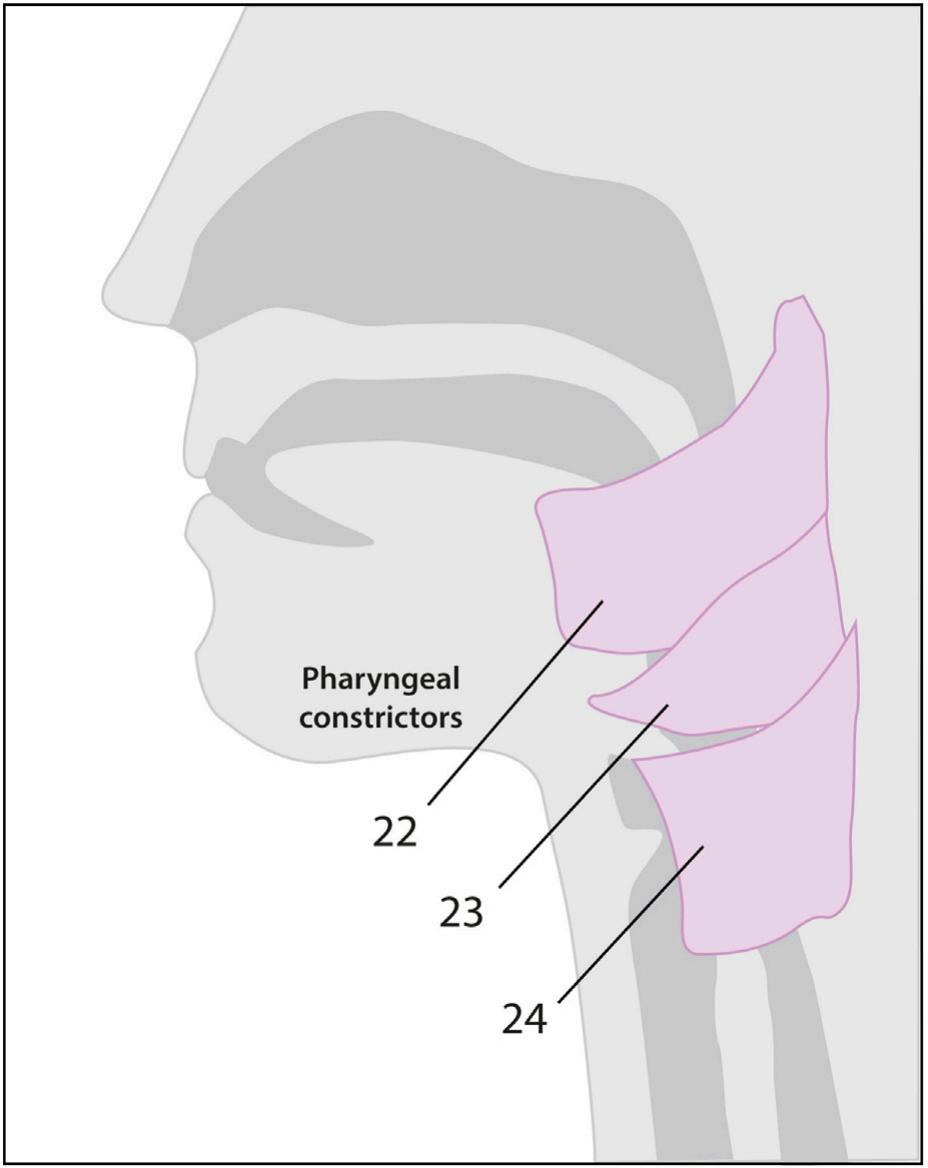	Peristalsis Open esophagus	Pharyngeal Constrictors 22. Superior 23. Middle 24. Inferior	Sphincters, push food into esophagus	X

**TABLE 2:** Schematic representation of sensory afferents and their actions in each phase of swallowing. Colors correspond to each cranial nerve and are similar to those in Table 1.

Phase	Cranial nerves	Action	Sensory function
Oral	V	Sense food in the mouth	Touch, pain, and temperature for face, oral cavity, teeth, and anterior tongue Proprioreception for jaw and teeth
	VII		Taste for anterior of the tongue
	IX		Taste, sensation for posterior of the tongue
Pharyngeal	IX	Sensation in the pharynx	Touch, pain, and temperature for upper pharynx
Esophageal	X	Esophagoglottal closure reflex	Touch, pain, and temperature for lower pharynx, larynx, and esophagus (including gag reflex)

**FIGURE 1:**
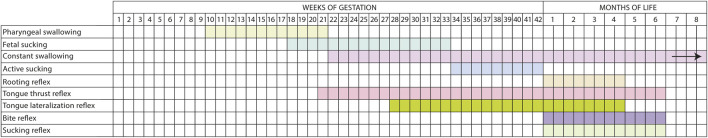
Relevant processes in the development of swallowing during gestation and the first few months of life. The swallowing reflex is preserved throughout life.

The development of the foundations of swallowing begins in the womb and progresses during infancy and the early years of childhood (Kalhoff et al., 2024). Starting from the 10th week of gestation, pharyngeal swallowing movements begin, and between the 18th and 24th weeks, fetal sucking movements develop (Figure 1). Consistent swallowing appears in the 22nd week, and active sucking emerges around the 34th to 37th weeks of gestation.

Both of these primitive motor reflexes (sucking and swallowing) are indispensable for he newborn´s survival (López Ortega et al., 2024; Rogers and Arvedson, 2005). They are involuntary reflexes which originate in the brainstem and disappear during early childhood development (although they may persist or reappear in neurological conditions as pathological signs of frontal release).

After 36 weeks of gestation, the maturation of the sucking-swallowing-breathing triad is complete, allowing healthy newborns to ingest liquid orally and swallow it (Kalhoff et al., 2024). The coordination of sucking, swallowing, and breathing is one of the most complex neuromotor programs in newborns, although the maturation of the stomatognathic system is not yet complete at birth (Palacio Ortega et al., 2024). Gradually, through sensorimotor learning, experience, neurological maturation, sensory integration, and desensitization of the gag reflex, the motor function of swallowing develops and is perfected, allowing for conscious and correct feeding (Kiyohara et al., 2012).

There are two types of swallowing: reflexive swallowing and voluntary swallowing. Reflexive or automatic swallowing refers to the swallowing of oropharyngeal secretions without the occurrence of the oral preparatory phase. It is unintentional and triggered by laryngopharyngeal sensory stimuli. It occurs unconsciously both when we are awake and during sleep. It has a weaker association with the corticobulbar tract and shows activation in the sensorimotor cortex, the lateral postcentral gyrus, and the right insula, although it generates greater activation in the left hemisphere (Kiyohara et al., 2012). It is a protective reflex action (to ensure the safety of the upper respiratory tract against any escape of food particles or saliva) and may also be related to emotions.

Voluntary swallowing occurs when a person has the desire to eat or drink while awake and conscious. It is part of feeding behavior, and there is an orderly activation of the muscles involved in each phase. Voluntary swallowing has a multiregional brain representation, stronger in the lateral sensorimotor and premotor cortex, the insula, the temporal cortex, the amygdala, the cerebellum, and the dorsal brainstem. It has a bilateral non-asymmetrical activation in both hemispheres, regardless of the subject’s lateralization (Kern et al., 2001).

**FIGURE 2:**
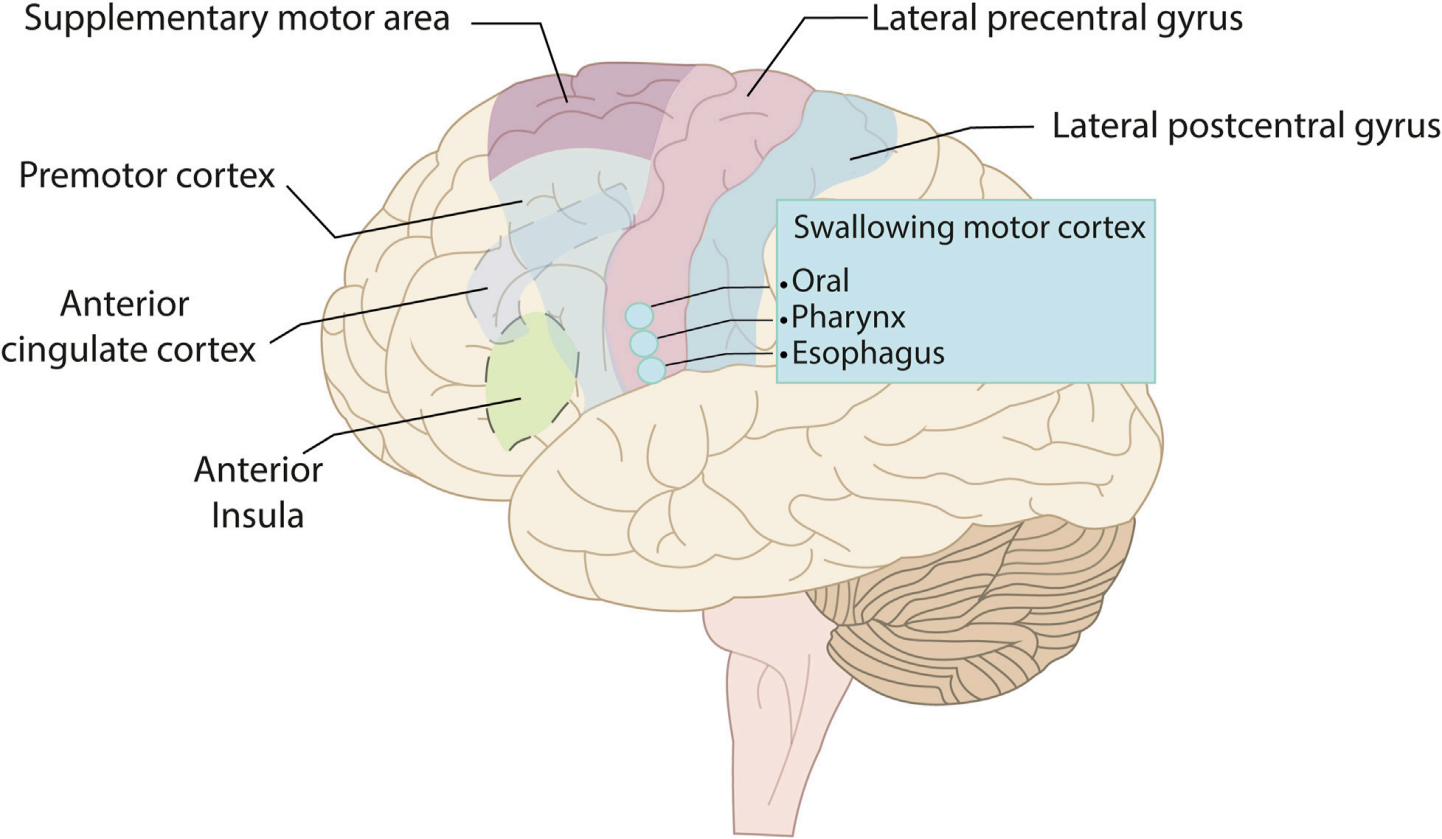
Lateral view of the left hemisphere showing the cortical areas involved in swallowing: The precentral gyrus, the swallowing motor cortex (located within the primary motor cortex), the premotor cortex, the supplementary motor area, the anterior cingulate cortex, the anterior insula, and the lateral postcentral gyrus.

Automatic and voluntary swallowing has been shown to be processed in areas of the cerebral cortex. Both types of swallowing are associated with the activation of several functionally distinct cortical areas in the prefrontal, temporal, and parietal lobes (Martin et al., 2001). The motor areas of the cerebral cortex that initiate spontaneous or voluntary swallowing have been recently identified. For many years, medical literature has stated that swallowing was primarily controlled by the brainstem (Penfield and Boldrey, 1937). However, more recent research has provided evidence that cortical and subcortical structures play a fundamental role in the control of swallowing, showing constant activity in multiple cortical areas (Figure 2): 1) The lateroventral precentral gyrus. In the face area (BA4), there is a somatotopic representation of the oral, pharyngeal, and esophageal muscles, with an asymmetric motor representation, independent of laterality (Hamdy et al., 1996). 2) The premotor cortex, with a focus of activation within the lateral premotor area (BA 6) (Wei et al., 2024). 3) The supplementary motor area would play an important role in mediating and preparing complex sequences of movements (Vasant and Hamdy, 2013). 4) The anterior cingulate cortex (BA24) is part of the limbic system and is important for processing motivation and attention towards voluntary actions and sensory stimuli (Vogt, 2005; Humbert and Robbins, 2007). 5) The lateral postcentral gyrus is where the primary somatosensory cortex of the face is located (Toogood et al., 2017). 6) The insula plays a role in mediating both sensory and motor aspects of the function of the alimentary tract involving the oropharynx, esophagus, and possibly other areas of the gastrointestinal tract (Binkofski et al., 1998; Deng et al., 2021).

### 2.1 Motor program of reflexive and voluntary swallowing

The initiation of the reflexive or automatic swallowing mechanism is triggered by the stimulation of sensory receptors located in the tongue, soft palate, uvula, posterior pharyngeal wall, and larynx. Voluntary swallowing is initiated by a direct action controlled by the cerebral cortex (Prosiegel and Ekberg, 2019).

**FIGURE 3:**
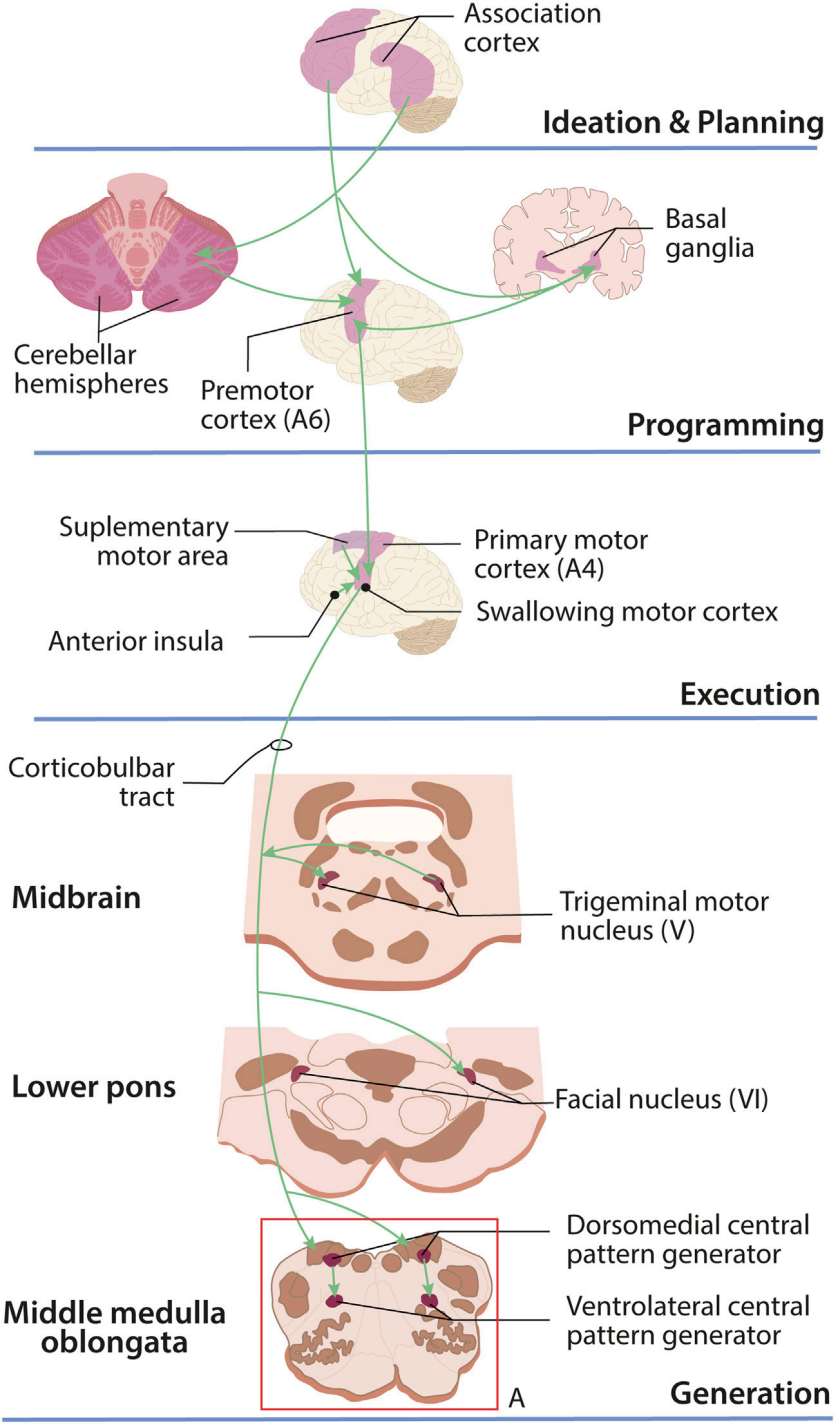
Motor control of swallowing in the central nervous system. The ideation of voluntary swallowing begins in the frontal cortex. Planning takes place in the prefrontal and somatosensory association cortices; planning is a joint effort between the cerebellum, motor cortex, and basal ganglia; for execution, the supplementary motor area, swallowing cortex, and insula send signals through the corticobulbar tract that will be received by the pattern generators (A) located in the medulla oblongata whose motor and sensory neurons will initiate and moderate swallowing.

The motor program of swallowing is divided into four stages (ideation and planning, programming, execution, and movement generation) and is a succession of excitatory and inhibitory mechanisms of motor nerve signals that are fed back by afferent sensory stimuli (Figure 3).

The ideation of voluntary swallowing originates in the frontal cortex. Movement planning occurs (following ideation in voluntary swallowing and in response to sensory stimuli in reflexive swallowing) in the prefrontal association cortex together with the somatosensory association cortex (Knollhoff et al., 2022). Upon receiving the signal, movement programming of the swallowing musculature is jointly performed by both cerebellar hemispheres and the basal ganglia, which work in parallel to program the movement of the swallowing musculature and then transmit this information to the premotor cortex, inhibiting the muscles that must remain relaxed.

For movement execution, the swallowing cortex (located in the anterolateral motor cortex) receives information from the premotor cortex and simultaneously receives impulses from the supplementary motor area and the anterior insula, which are sent via the corticobulbar tract (Li-Jessen et al., 2020). Swallowing movements are produced by central pattern generators (CPGs) located in the medulla oblongata, which receive signals via the corticobulbar tract.

The swallowing network includes two main CPGs (Figure 3A). A dorsal CPG containing the generator neurons involved in triggering, shaping, and synchronizing the sequential or rhythmic swallowing pattern. It is located in the dorsal medulla oblongata, within the nucleus of the solitary tract and the adjacent reticular formation. The second CPG is ventrolateral and distributes motor outputs to cranial nerves V and VII located in the pons and to cranial nerves IX, X, XI, and XII in the medulla oblongata. This latter CPG is located in the ventrolateral medulla oblongata, in the reticular formation surrounding the nucleus ambiguous (Pitts and Iceman, 2023).

**FIGURE 4:**
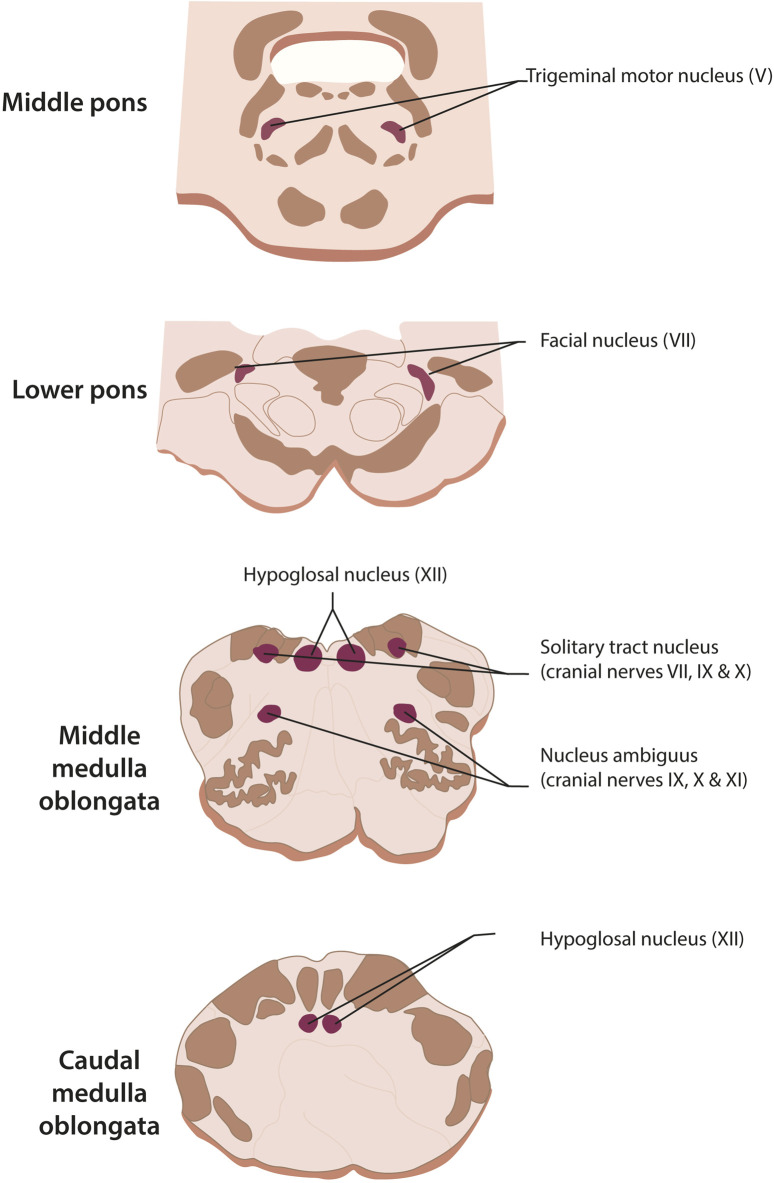
Cranial nerve nuclei which are involved in swallowing. Motor nuclei: nucleus ambiguus (cranial nerves IX, X, and XI, the latter not involved in swallowing), trigeminal motor nucleus (for V), facial motor nucleus (cranial nerve VII), and hypoglossal nucleus. Sensory nuclei: nucleus of the solitary tract (cranial nerves VII, IX, and X) and specific regions of the trigeminal nucleus (cranial nerve V).

The corticobulbar tract also sends information directly to the four nuclei of the cranial nerves involved in swallowing (Figure 4): a) The motor nucleus of the trigeminal nerve (V) b) The facial motor nucleus (VII). c) The hypoglossal nucleus (XII) and d) The nucleus ambigus, with cranial nerves IX, X, and XI (the latter is not involved in swallowing). Two sensory nuclei are also involved, which receive information from peripheral structures: a) the nucleus of the solitary tract (with cranial nerves VII, IX, and X) and b) specific regions of the trigeminal sensory nuclei (V). Sensory information has three functions: helping to initiate swallowing, modifying the threshold for pharyngeal swallowing, altering the level of muscle recruitment during swallowing, recognizing the characteristics of the stimulus (Wei et al., 2024).

### 2.2 Phases of swallowing

The four phases of swallowing are coordinated with each other through central pattern generators (CPGs) and peripheral reflexes. Signals generated by CPGs must be amplified by peripheral sensory afferents that feed back onto the CPGs, producing each specific phase of swallowing (Lang, 2009). The first phase of swallowing is the oral phase. Within this phase, two phases can be identified: the preparatory and the transport phase. The oral preparatory phase is voluntary, and it is where mastication and bolus formation take place. The airway is normally open, and the duration of this phase depends on factors such as motor efficiency and the subject’s desire to savor the food (Ertekin and Aydogdu, 2003). The oral transport phase begins with the transport of the bolus towards the pharynx. The tongue movement presses the bolus against the hard palate and directs it backwards. By the contraction of the hyoglossus muscle, the tongue adopts a channel shape, the palatoglossal seal opens and allows the food to advance. This phase is voluntary, and its duration depends on the viscosity and size of the bolus and the state of the higher brain functions. The oral phase ends when the food bolus exerts pressure on the anterior palatal arches, the back of the tongue, and the palate (Costa, 2018).

The pharyngeal phase is the set of processes that occur between the passage through the isthmus of the maw until the bolus passes through the upper esophageal sphincter. This phase is reflexive, lasts approximately 1 s and is initiated by the stimulation of pharyngeal mechanoreceptors and tongue proprioceptors that send information to the CNS. In this phase, four events occur: a) closure of the velopharyngeal sphincter, b) closure of the laryngeal sphincter, c) propulsion of the bolus through the pharynx and d) opening of the UES, transforming the oropharynx into a swallowing pathway (Rodríguez and Belinchón, 2020).

From the moment the bolus passes through the upper esophageal sphincter (UES), the esophageal phase begins and ends when the bolus reaches the stomach, thanks to the peristaltic wave produced by the esophageal muscle layers. The lower esophageal sphincter (LES), which is physiological and not anatomical, opens due to the presence of the bolus and the esophageal peristalsis itself, allowing entry into the stomach after which it recovers its tone, preventing gastroesophageal reflux. It has a duration of between 8 and 20 s and is also involuntary (Costa, 2018). The interruption or deterioration of any phase of the swallowing mechanism can compromise the safety and effectiveness of it. Each of the processes must be functionally evaluated to identify the physiological or structural cause that produces swallowing dysfunction.

## 3 Dysphagia

Dysphagia is the difficulty in moving food or liquids from the mouth to the stomach, due to the impairment of one or more phases of swallowing, which alters its efficiency and safety (McCarty et al., 2021). Moreover, its efficiency is compromised when the individual is unable to carry out the processes of eating correctly, which may result in malnutrition and/or dehydration. The alteration of safety (aspiration or penetration into the airway) results in respiratory infections, with aspiration pneumonia being the cause of death in 50% of cases (Hurtte et al., 2023). It is estimated that dysphagia affects 8% of the world’s population, and its incidence increases in the elderly population (Nagano et al., 2023). In older adults living independently, the prevalence of dysphagia is between 30% and 40% and increases to 60% in institutionalized elderly patients. In elderly patients with neurological disorders and/or dementia, the prevalence increases to 64% and 80%, respectively (Baijens et al., 2016). Additionally, swallowing disorders produce a series of medical, social and psychological sequelae that can lead to malnutrition, dehydration, pneumonia, chronic lung disease and decreased quality of life (Milewska et al., 2020). Fear of choking, changes in food, eating and drinking slowly, fatigue and embarrassment about eating in public affect participation in social events. The preparation of special diets can also increase the financial burden and stress of caregivers and families (Smith et al., 2023).

### 3.1 Types of dysphagia

Based on the affected swallowing phase, dysphagia can be classified into oropharyngeal dysphagia (oral and pharyngeal phases) and esophageal dysphagia (esophageal phase). Oropharyngeal dysphagia is the most prevalent one and is further divided into two disorders of two of the phases, the oral and pharyngeal (Malhi, 2016). Oral dysphagia presents signs such as difficulty initiating swallowing, problems forming the bolus and controlling it within the oral cavity. Prolonged bolus retention is associated with drooling, sialorrhea, and the need for multiple swallowing attempts. Oral dysphagia can be caused by incomplete lip closure, reduced strength or incompetence of the mastication muscles, and/or a limitation or incoordination of tongue movement (Rech et al., 2018). Pharyngeal phase dysphagia is usually due to problems with lingual, pharyngeal propulsion, or opening of the upper esophageal sphincter. This phase is under involuntary control and the alterations that may occur are: delayed swallowing reflex, decreased velopharyngeal closure (with nasal regurgitation), decreased epiglottic movement, poor laryngeal elevation, and disorders or lesions of the upper esophageal sphincter. These alterations cause bolus retention in the pharynx, which can lead to coughing, nasopharyngeal regurgitation, sialorrhea or xerostomia, fragmented swallowing, dysarthria, choking, and even aspiration or penetration into the airway (Roy et al., 2007). Esophageal dysphagia (ED) presents as difficulty continuing to swallow and may be associated with chest pain or pressure, reflux, food regurgitation, repeated swallowing attempts, and symptoms of aspiration, such as coughing and choking. The main cause of ED is inflammation or stenosis of the esophageal wall. Neurological diseases can also cause alteration of the smooth or striated muscle of the esophagus, or of the motor neurons that control esophageal peristalsis and relaxation of the lower esophageal sphincter (Louis and Nakhal, 2022).

### 3.2 Causes of oropharyngeal dysphagia

**FIGURE 5:**
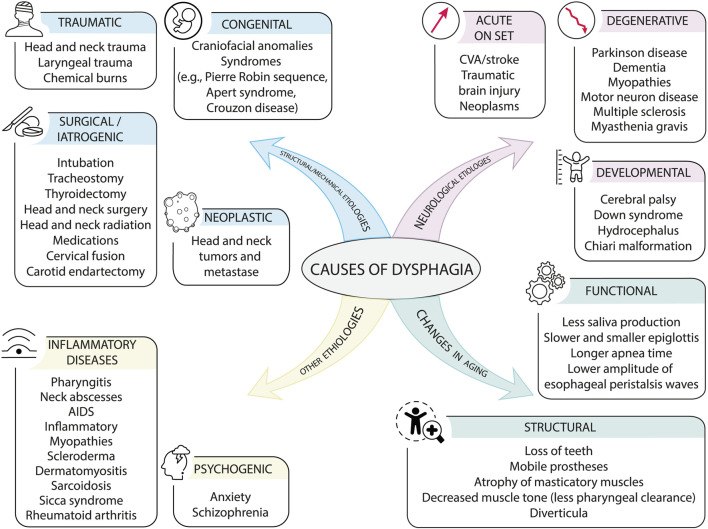
Causes of dysphagia. Causes of dysphagia can be divided into neurological, structural, other etiologies, and age-related changes, although the latter may not be a cause of dysphagia but rather an early symptom.

Oropharyngeal dysphagia (OD), accounting for 80% of all diagnosed dysphagias, is a symptom rather than a disease itself. Any abnormality of the critical oropharyngeal structures involved in swallowing or of the neural controls can impair swallowing and cause OD. The causes of OD can be grouped into three categories (Figure 5): neurological, structural, and other etiologies (Wilkinson et al., 2021). Ageing itself is not a cause of dysphagia, although there are age-related changes that affect all phases of swallowing (González Cortés et al., 2018). Neurological causes affect the transmission of information to and from the CNS. Consequently, the function of the oropharyngeal neuromuscular system is altered. These causes can be divided into three types: a) Acute onset: such as stroke, traumatic brain injury, and neoplasms. b) Neurodegenerative: Parkinson’s disease, dementia, myopathies, motor neuron disease, multiple sclerosis, and myasthenia gravis. c) Developmental: cerebral palsy, Down syndrome, hydrocephalus, and Chiari malformation (Malandraki and Robbins, 2013).

Mechanical and structural causes are those that alter the anatomical and/or physiological integrity of the oropharyngeal mechanism and cause difficulty in swallowing. They are divided into: a) congenital causes, such as craniofacial anomalies and/or syndromes (e.g., Pierre Robin sequence, Apert syndrome, Crouzon disease). b) traumatic causes, including head and neck trauma, laryngeal trauma, and chemical burns. c) surgical or iatrogenic causes: intubation, tracheotomy, thyroidectomy, head and neck surgery and radiotherapy, medication, cervical fusion, carotid endarterectomy. d) Neoplastic causes such as tumors and head and neck cancer (Rommel and Hamdy, 2016).

Finally, there are other causes of dysphagia, including inflammatory diseases such as pharyngitis, cervical abscesses, AIDS, inflammatory myopathies, scleroderma, dermatomyositis, sarcoidosis, Sjögren’s syndrome, rheumatoid arthritis, and psychogenic disorders such as anxiety and schizophrenia (Massa et al., 2022). In patients under 60 years of age, morphological and structural causes predominate, while in patients over 60 years of age, neurological alterations are the most frequent (Tulunay-Ugur and Eibling, 2018).

Depending on the underlying cause and the affected swallowing phase, dysphagia can manifest in different ways. In the oral phase, problems may be associated with food crushing, abnormal chewing, poor bolus formation and transport, poor tongue mobility, poor or absent lip closure, excessive salivation, numbness of the mouth, and/or dry mouth. As a result, food may accumulate in the vestibule of the mouth and in the cheeks, causing saliva leaks or food leakage from the mouth (Dylczyk-Sommer, 2020).

A consequence of the severity of pharyngeal dysphagia, regardless of its cause, is that the protective function of the larynx is altered: the cough reflex is weakened, triggering the passage of food or secretions into the airway. The consequences of airway invasion can be retention, penetration, and/or aspiration of saliva or food. Retention is defined as the accumulation of saliva or food in the vallecula or pyriform sinuses. Penetration occurs when saliva or food enters the laryngeal vestibule, and aspiration is associated with the presence of saliva or food below the vocal cords, being the most severe of the three (Speyer et al., 2022).

### 3.3 Assessment of oropharyngeal dysphagia

When a patient or their family notices difficulties with swallowing, they inform the doctor, who carries out an assessment of OD. The aim of the assessment is to determine the efficacy and safety of swallowing. Efficacy is demonstrated when the patient is able to ingest all the calories and water, they need to be well-nourished and hydrated. Swallowing is safe when the individual is able to ingest food and drink without respiratory complications arising (Alsaied and Postma, 2019). There are two types of assessments to achieve this goal: non-instrumental or clinical assessment and instrumental assessment, which is carried out using complementary tests with specific tools.

#### 3.3.1 Non-instrumental assessment

Non-instrumental assessment of dysphagia is effective for: 1) identifying the potential causes of swallowing problems; 2) estimating the safety of swallowing and the risk of aspiration; 3) supporting decisions about oral feeding or alternatives; and 4) deciding on treatment strategies and identifying the need for additional instrumental assessment (Farneti et al., 2018). Clinical assessment encompasses several aspects, and, in the absence of a systematic review, five types of necessary examinations can be established: patient medical history; assessment of cognition and communication; physical examination; administration of a screening test; evaluation of oral intake with different textures; and observation of oral hygiene (Clavé et al., 2008). During medical history, sociodemographic data, health information, and comorbidity and other risk factors for dysphagia should be collected. An initial observation of the level of consciousness and degree of postural control should also be made, as well as whether the patient is able to sit up and remain upright. The physical examination of oral function includes the motor and structural examination of all oral functions, including examination of the cranial nerves and the velopharyngeal, cough, and swallowing reflexes (Clavé et al., 2011).

Once the medical history, physical examination, and assessment of cognitive status have been carried out, it is necessary to know the patient’s perception of their disorder and their quality of life. The most used subjective screening questionnaires for dysphagia are the Swallowing Quality of Life Questionnaire (SWAL-QOL) and the Eating Assessment Tool (EAT-10), both validated and very simple to apply. The SWAL-QOL consists of 44 items and provides information on self-perception of quality of life and the impact of swallowing on the patient (McHorney et al., 2002). The EAT-10 assesses ten items and can be answered by patients or their caregivers. Statements about the experience of swallowing are rated on a scale of 0–4, where 0 indicates no problem and 4 indicates a severe problem. A score greater than 3 indicates that the individual is at high risk of dysphagia (Sánchez-Sánchez et al., 2021; Zhang P. et al., 2023).

To complete the clinical assessment and make it useful for diagnosis, an oral intake assessment must be performed. The most commonly used methods of assessing oral intake are: the water test, the Gugging Swallowing Screen (GUSS), and the Volume-Viscosity Swallow Test (V-VST) (Clavé et al., 2015). The water test is the most widely used in nursing. In the test, 10 mL of water is administered to the patient and the presence of the following symptoms is observed: drooling, number of swallows, coughing, and dysphonia (Depippo et al., 1992). This is repeated four times with the same volume of water (10 mL) and ends with the administration of 50 mL of water in a glass. The test is positive if any symptom is present and negative if none are present. The drawback of this test is that it is only performed with large volumes, a single texture (water), and uses coughing as the sole sign of detecting aspiration. In patients with an altered cough reflex and/or low pharyngeal sensitivity, it will not be possible to detect the existence of silent aspirations and/or penetrations.

The GUSS test is divided into 2 parts: a preliminary or indirect assessment (swallowing saliva) and the direct swallowing test, with 3 different textures (semi-solids, liquids, and solids), which are performed sequentially. In the direct swallowing test, swallowing, involuntary coughing, drooling, and voice change are monitored. In the indirect test, alertness is added. Each observation is quantified by points, from 0 to 5, assigning 0 points to pathological swallowing, 1 point to delayed swallowing, and 2 points to normal swallowing. Patients evaluated must score at least 5 points in each test; if not, the test is stopped due to a high risk of aspiration. The GUSS test is validated, although the texture variation is limited (pudding, liquid, and solid) (Trapl et al., 2007).

The V-VST involves administering 5, 10, and 20 mL of food in nectar, pudding, and liquid textures, prepared with commercial thickener and in a pre-established order. It always starts with a safe bolus for the patient of 5 mL of nectar viscosity. The following signs should be observed: presence of cough, vocal changes, oral residue, fragmented swallowing, incompetence of lip closure, and pharyngeal residue. While performing the test, oxygen saturation is monitored. A decrease in basal O2 saturation ≥3% is a sign of aspiration. If, during the test, the patient presents alterations in any of the observed signs, the volume and viscosity evaluated at that time will be considered an unsafe bolus for the patient, so it will be necessary to increase the viscosity and/or decrease the volume to be able to continue with the test (Zugasti Murillo et al., 2023; Zhang P. et al., 2023). The diagnostic sensitivity of the V-VST for alterations in the safety and efficacy of swallowing is 88.1% and 89.8%, respectively. This test also helps determine the safest and most effective bolus volume and viscosity for the patient’s oral intake.

#### 3.3.2 Instrumental assessment

A patient who, after the clinical assessment, has shown signs of dysphagia, should undergo a complementary instrumental examination with tools that help to diagnose the functional disorder. Instrumental assessment techniques include: fiberoptic endoscopic evaluation of swallowing (FEES), videofluoroscopy (VFS), and high-resolution esophageal manometry (HRM). Each has advantages and limitations. These tools allow for the acquisition of images and objective data on the swallowing process (Scottish Intercollegiate Guidelines Network, 2010).

FEES is performed with a flexible fiberoptic scope connected to a light source through the nasal cavity to the nasopharynx. It provides anatomical information of the larynx and vocal cord function and allows assessment of the location of secretions and the patient’s ability to clear them. The assessment can also be performed with foods of different textures and volumes, thus assessing the passage of the bolus to the hypopharynx, aspiration, penetration, and the presence of residue (Triggs and Pandolfino, 2019).

In VFS, equipment is used that provides a real-time visualization of the anatomy and physiology of swallowing, along with different consistencies of barium sulphate prepared with commercial thickener. It is the gold standard for assessing OD and swallowing safety (Giraldo-Cadavid et al., 2017). This assessment allows for identification of failures in the safety and efficacy of swallowing, quantification of the oropharyngeal motor response, measurement of hyoid movement, opening of the UES, and establishment of the presence of silent aspiration (without the cough reflex) (Helliwell et al., 2023). Since barium aspiration can pose safety problems, water-soluble contrast agents are usually the preferred option. On the other hand, it must be ensured that the benefits of exposure to ionizing radiation outweigh the risks to which the patient is exposed (RCSLT. Videofluoroscopic evaluation of oropharyngeal swallowing function VFS, 2013). The inability to transport the VFS equipment and the impossibility of performing the test in people with low consciousness limit its use.

HRM is performed with a flexible catheter with sensors (one per centimeter), which is introduced through the nostrils, through the esophagus and into the stomach. The pressure data from these sensors are presented as a colour-coded topographic graph of esophageal pressure activity (Yazaki et al., 2024). HRM provides a simultaneous and integrated assessment of pharyngeal, esophageal, upper esophageal sphincter, and lower esophageal sphincter function in real time. It is considered the gold standard for identifying and classifying esophageal motility disorders (Fox et al., 2021).

## 4 Ageing and presbyphagia

Presbyphagia, also known as ‘primary dysphagia’, is the result of the effects of the normal ageing process on the swallowing organs in healthy older adults (Martin et al., 1991). It is understood as a transitional state between healthy swallowing and oropharyngeal dysphagia, resulting from all the anatomical and functional changes that occur with ageing, be they central and/or peripheral (Figure 4) (Ambiado Lillo and Borjas Galvis, 2021; Labeit et al., 2022).

### 4.1 Changes in swallowing physiology associated with ageing

**FIGURE 6:**
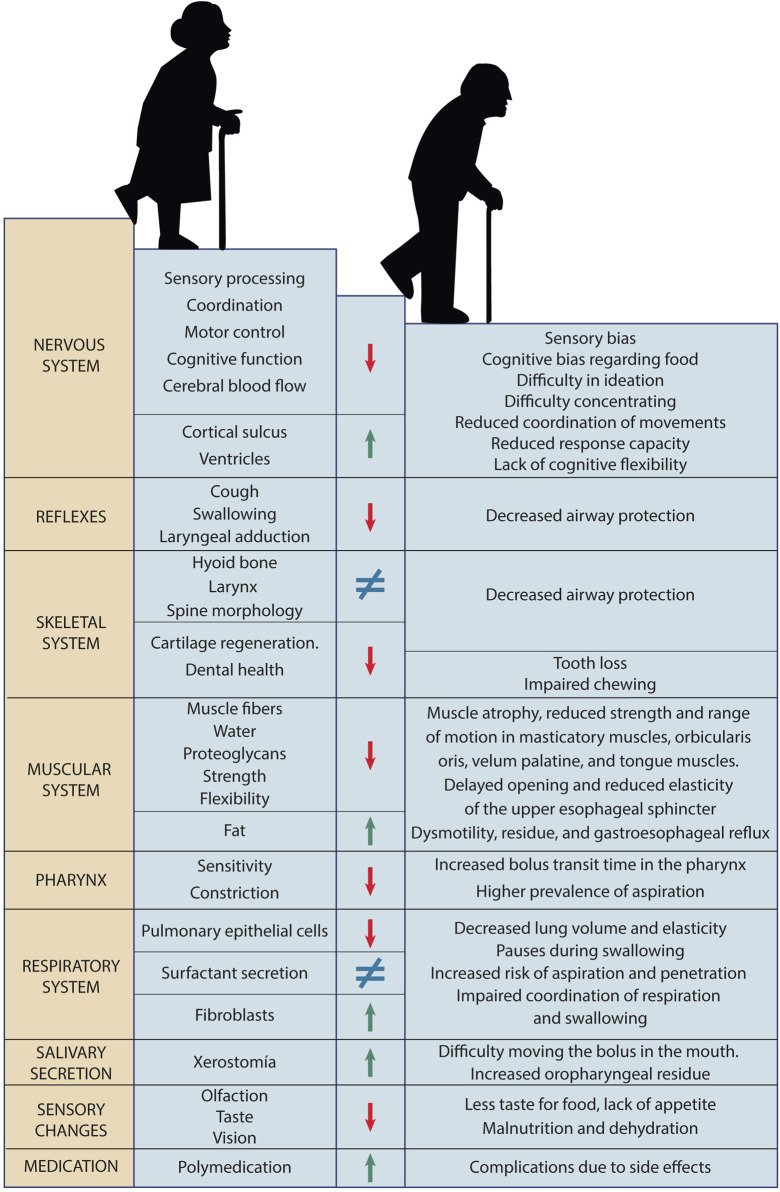
Changes during aging. Affected system, structures and functions affected, improvement or worsening, and impact on swallowing.

During ageing, physiological functions gradually deteriorate. This functional degradation is caused by nine molecular and cellular phenomena associated with normal ageing, classified as primary, antagonistic, and integrative (Hou et al., 2019). Primary changes include genomic instability, telomere attrition, epigenetic alterations, and loss of proteostasis. Antagonistic features (mitochondrial dysfunction, cellular senescence, and deregulated nutrient sensing) are compensatory responses to primary damage but can become detrimental over time. Integrative features (stem cell exhaustion and altered intercellular communication) arise as a result of the cumulative damage produced by primary and antagonistic features and would be responsible for the functional decline associated with ageing (López-Otín et al., 2023). The manifestation of these processes affecting swallowing are changes in the nervous, skeletal, muscular, sensory, and respiratory systems (Figure 6).

**FIGURE 7:**
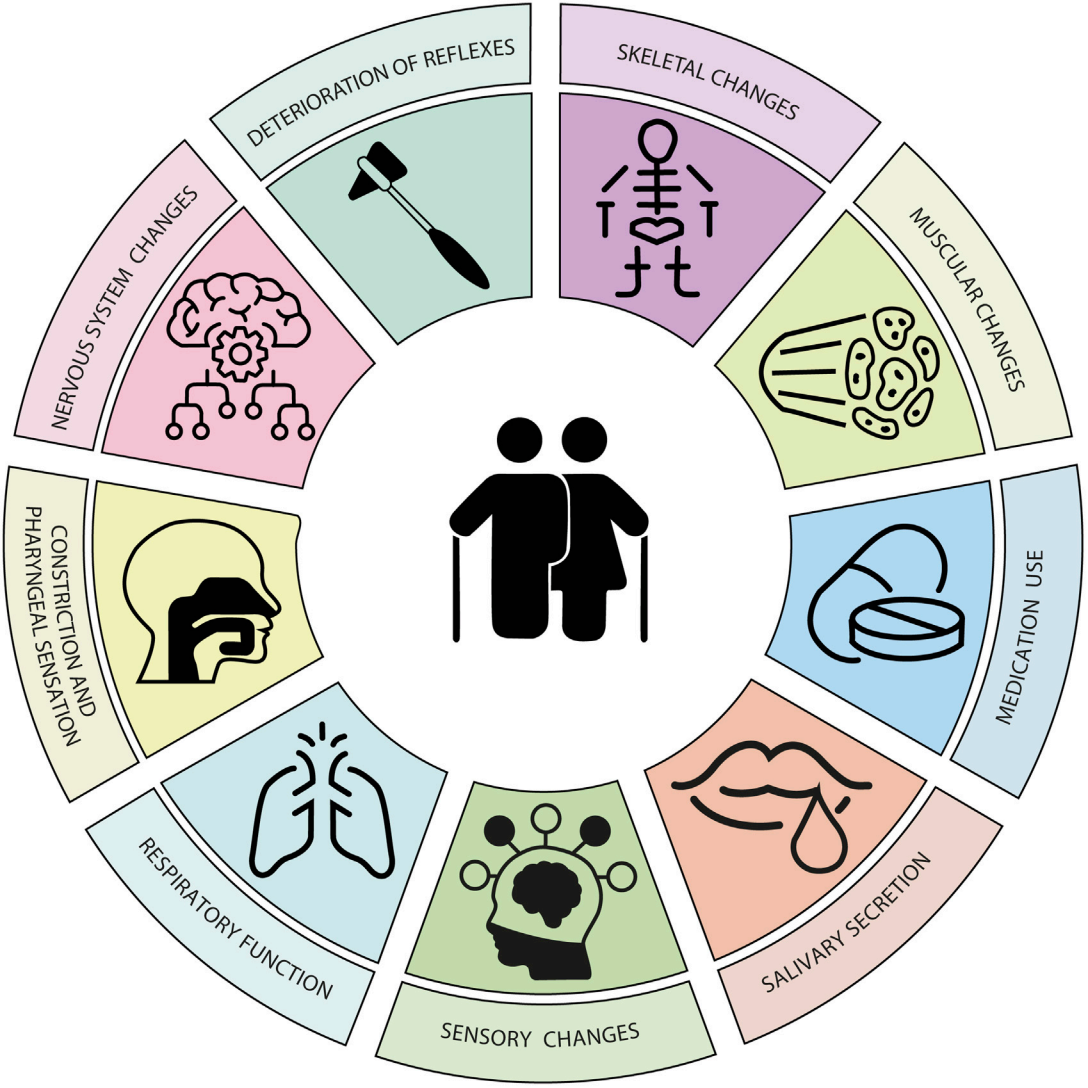
Age-related changes affecting swallowing. The combination of skeletal, muscular, sensory, and respiratory changes, along with decreased reflexes, salivation, laryngeal constriction and sensation, and increased medication use in this stage of life contributes to the onset of presbyphagia.

Neurodegeneration during ageing decreases activation of areas corresponding to sensory processing, sensorimotor integration, coordination, and motor control (Figure 7). The volume of some neuronal nuclei and the capacity to process information decrease. As anatomical manifestations of changes in the nervous system, cerebral blood flow is reduced, the cortical sulcus widens, and the ventricles enlarge, and dysmotility associated with a decrease in myenteric neurons of the esophagus occurs (Feng et al., 2023). Moreover, the decrease in cognitive function in ageing causes cognitive bias regarding food characteristics, difficulty in ideation and concentration. Older adults have a lower response capacity, poor coordination of movements, and lack of flexibility to adjust the speed of eating and food intake (Harada et al., 2013). Changes in the nervous system during ageing often also lead to a deterioration of swallowing reflexes, the cough reflex (which protects the airways), and laryngeal adduction (Katsumata et al., 1995; Ebihara et al., 2012).

The skeletal system involved in swallowing is also affected by the changes of ageing. The positions of the hyoid bone and larynx are altered due to variations in the morphology of the spine and the reduction of muscle tone (Kang et al., 2010). Aged cartilage (due to the accumulation of senescent chondrocytes) changes shape and becomes less elastic, weakening the protective capacity of the airways of the epiglottis and arytenoid cartilages (Williams and Day, 2003; Coryell et al., 2021). With ageing, the neurovascular structures of the dental pulp are also affected and teeth become sensitive, painful, when injured and infected. Both the loss of teeth and the use of poorly adapted prostheses negatively influence swallowing (Bartlett and O’Toole, 2019).

Total muscle mass in older adults, water content in muscles, and proteoglycans in tendons decreases, while fat accumulation increases (Larsson et al., 2019). This causes a decrease in muscle contraction force, with less flexibility and less coordination in swallowing functions (Dao et al., 2020). The orbicularis oris muscle (causing leakage), the masticatory muscles, the velum palatine, and the muscles of the tongue are affected, showing muscle atrophy, reduced strength, and reduced range of motion (McCoy and Desai, 2018). Similarly, the number of muscle fibers of the UES decreases and the impulses that maintain cricopharyngeal tension also gradually decrease. This results in a delayed opening time and less amount of UES openings. On the other hand, muscle connective tissue increases, which generates less elasticity and distensibility of the sphincter. The degree and duration of UES opening are abnormal, which increases the risk of aspiration, pharyngeal residue, and gastroesophageal reflux (Cock and Omari, 2018).

The impairment of respiratory function with ageing is caused by the decrease in the number of pulmonary epithelial cells, the increase in the proportion of fibroblasts, and the alteration of surfactant. All this causes a decrease in lung volume and elasticity, reducing the ability of older adults to clear respiratory waste, increasing the risk of aspiration and penetration (Brandenberger and Mühlfeld, 2017). The weakening of respiratory function causes older adults to take longer pauses in breathing during swallowing and this compromises the coordination of both functions (Skloot, 2017).

Sensory perception also progressively deteriorates with ageing. Older adults have thinner oral mucosa, weaker secretion, fewer chemoreceptors, and reduced taste perception. This reduction in smell and taste can lead to a decreased appetite and malnutrition or dehydration (Lin, 2020). Decreased pharyngeal constriction and sensation prolongs bolus transit time through the pharynx and increases the prevalence of post-swallow aspiration (Namasivayam-MacDonald et al., 2018). The prevalence of xerostomia or dry mouth increases with age. Reduced saliva production affects food propulsion in the mouth, increases oropharyngeal residue, and the risk of poor oral hygiene (Toan and Ahn, 2021). When combined with the high consumption of medications by older adults, there is a greater risk of adverse effects on swallowing function.

Although swallowing is often preserved with age, it can exhibit signs of deterioration due to physiological changes, which worsen as the ageing process progresses. Most of these abnormalities are usually clinically silent because the patient compensates intuitively and effectively by changing the consistency of the diet and the duration of feeding (Dominguez et al., 2022; Cavallaro et al., 2022). In the presence of weakness or acute illness, a person with undiagnosed presbyphagia or aged swallowing can transition to oropharyngeal dysphagia (Namasivayam-Macdonald and Riquelme, 2019).

## 5 Treatment of dysphagia

**FIGURE 8:**
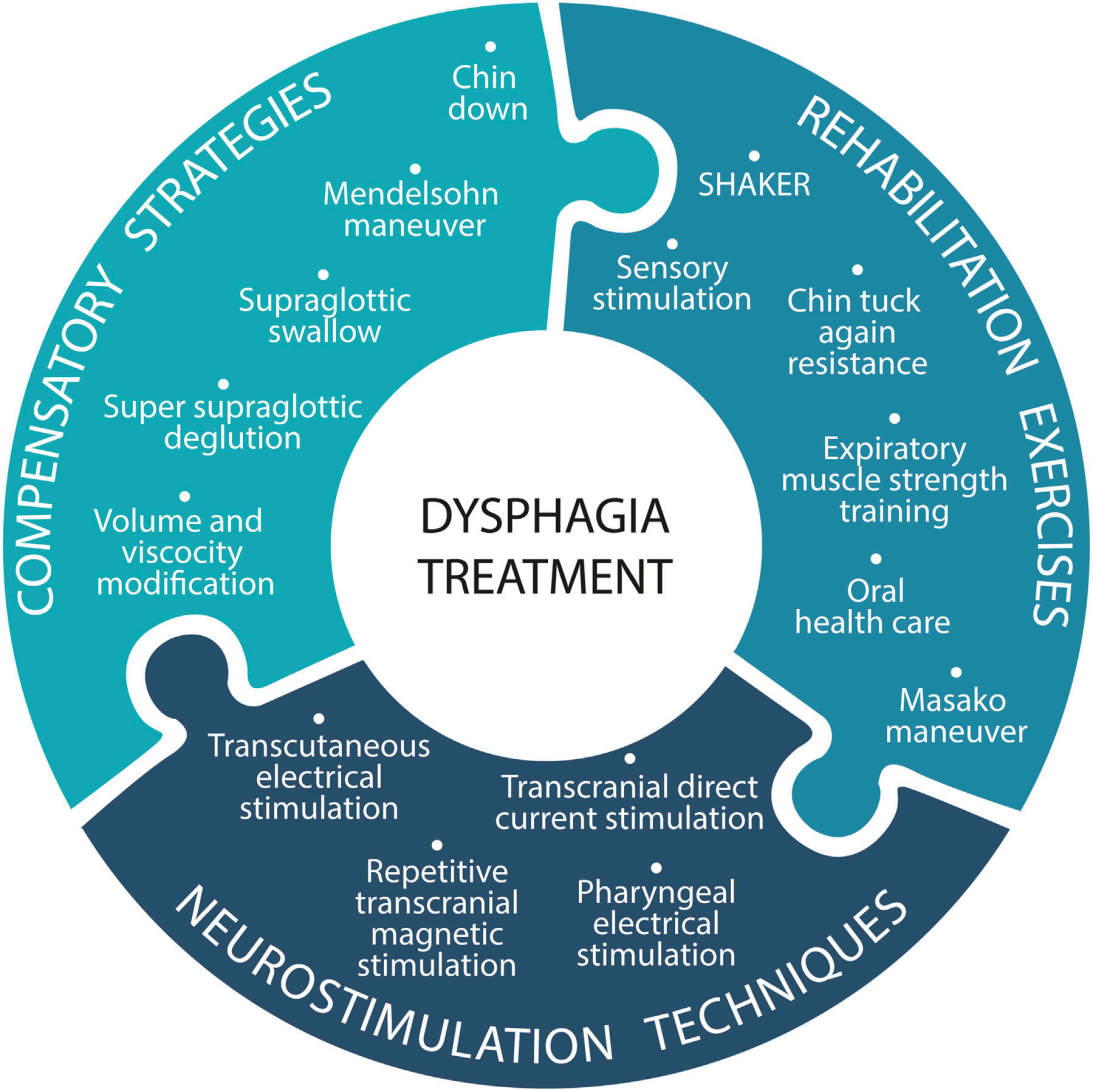
Swallowing treatments: a range of compensatory strategies, rehabilitation exercises, and neurostimulation techniques can be used alone or in combination.

Over the past 2 decades, treatments for dysphagia have evolved from 1) compensatory strategies to 2) rehabilitation strategies and subsequently to 3) neurostimulation techniques that facilitate neuroplasticity for functional recovery (Figure 8). Compensatory interventions are used to modify body postures and/or bolus characteristics to reduce the risk of aspiration. The most commonly used interventions are the chin-down posture, the Mendelsohn maneuver (voluntary prolongation of the hyolaryngeal elevation and the opening of the upper esophageal sphincter), the supraglottic swallow, and the super-supraglottic swallow (to close the vocal cords and supraglottic structures) (Calandra-Buonaura et al., 2021). Food and liquid modifications are made after assessing the safe volume and viscosity for the patient. Commercial thickeners are best suited for modifying food textures, and gelled water is recommended for safe ingestion of liquids (Newman et al., 2016).

Rehabilitation exercises aim to restore swallowing function. These include: head lift or Shaker exercise and chin tuck with resistance (CTAR) to strengthen the suprahyoid muscles, increase hyoid bone movement during swallowing, and increase the opening of the upper esophageal sphincter (Park and Hwang, 2021); the Masako maneuver or tongue-holding swallow to strengthen the base of the tongue and pharyngeal wall movement (Balou et al., 2019); expiratory muscle strength training (EMST) to synchronize breathing and swallowing and improve protective coughing (Pauloski and Yahnke, 2022); and sensory stimulation with temperature and taste contrasts. Poor oral health, in combination with dysphagia, has been identified as a dominant risk factor for aspiration pneumonia. It is believed that aspiration of saliva contaminated with bacteria is the primary pathogenic mechanism of pulmonary infections in patients with dysphagia. For this reason, oral healthcare is a supplementary recommendation for any dysphagia treatment (Dziewas et al., 2021).

Both peripheral and central (cortical and cerebellar) neurostimulation treatments can promote neuroplasticity in patients with dysphagia by modulating synaptic strength. Peripheral neurostimulation is primarily used to increase sensory information to the central nervous system, while central brain stimulation is used to induce plasticity changes in the cortex and/or cerebellum directly (Cheng and Hamdy, 2022). Neurostimulation techniques include transcutaneous electrical stimulation (TES) (Miller et al., 2022), repetitive transcranial magnetic stimulation (rTMS) (Sheng et al., 2023), transcranial direct current stimulation (tDCS) (Bengisu et al., 2024), both for cortical areas, and pharyngeal electrical stimulation (PES) (Labeit et al., 2024), targeting the pharyngeal sensory and motor cortices in the peripheral sensory afferent system. Transcutaneous electrical stimulation is used to activate the sensory nerves (sensory transcutaneous electrical stimulation) or the muscles (neuromuscular electrical stimulation) involved in swallowing function by stimulating the axonal motor nerve endings and muscle fibers (Pauloski and Yahnke, 2022).

Modified PES induces a higher frequency of involuntary swallowing. Activations of cortical regions related to swallowing have been observed and resulted in greater efficiency of the swallowing process after prolonged intervention. Therefore, it is believed that modified pharyngeal electrical stimulation could reorganize the neural network related to swallowing, making it a promising neurostimulation therapeutic method for severe neurogenic dysphagia (Zhang X. et al., 2023). Thus, the results obtained with neurostimulation techniques, including non-invasive brain stimulation and stimulation methods targeting the afferent neural pathways of swallowing, are positive. However, there is inter-subject variability in the responsiveness to these treatments (Simons and Hamdy, 2017). Genetic predisposition, brain configuration, and the level of neuronal activation prior to neurostimulation are some of the factors that may influence this variability (Rosenbek, 2014).

### 5.1 Neuroplasticity and neurorrehabilitation

Ramón y Cajal was one of the first to suggest that the nervous system has an inherent ability to adapt and reorganize itself in response to experience, learning, and new physiological or even pathological conditions that arise during development or as a result of its interaction with the environment (Ramón y Cajal, 1909). Neuroplasticity or neural plasticity refers to the changes that occur in neural pathways to enhance their function through synaptogenesis, reorganization, strengthening, and suppression of networks (Pauloski and Yahnke, 2022). An example of neuroplasticity is the increase in the number of branches and synapses in older adults, thus compensating for the loss of neurons that occurs throughout life. It has been shown that the dendritic arborizations of pyramidal neurons in the cerebral cortex are 25% longer in healthy 80-year-old individuals than in 50-year-old subjects (Portera Sánchez, 2002).

The objectives of rehabilitation processes after the loss of a previously acquired function are the reconnection of neural networks and the development of new connections between neurons. Cerebral neuroplasticity not only functions as an automatic mechanism but also occurs when external stimuli are perceived, new ideas are generated, different emotions are felt, or newly learned movements are performed correctly (Saway et al., 2024). In this way, when neuroplasticity is used as a therapeutic tool, new neural connections are generated, which will form the basis for new learning (Kolb and Muhammad, 2014). This idea is fundamental to understanding how swallowing function can be recovered after a neurological injury, such as a stroke.

**FIGURE 9:**
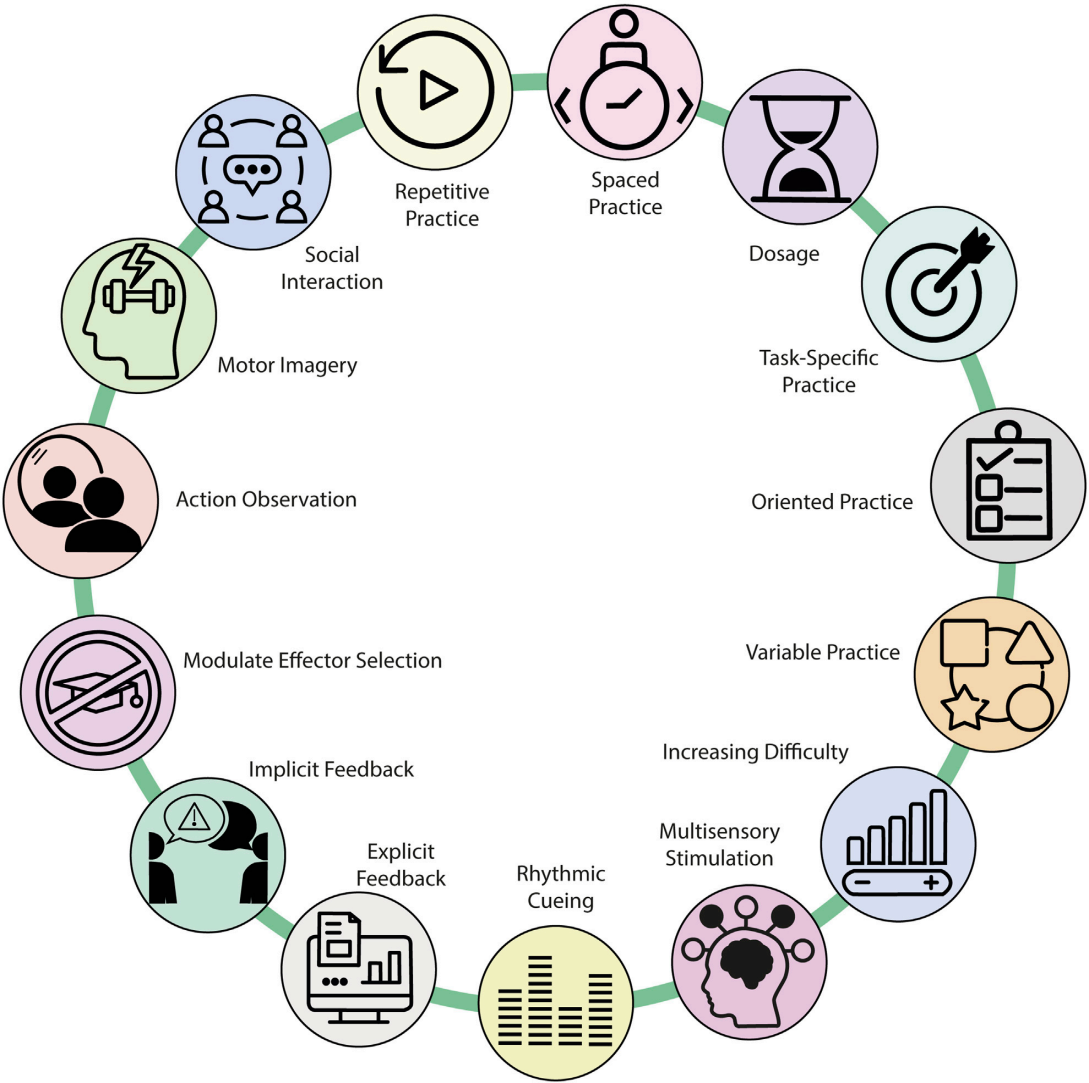
Neuroplasticity-driven principles of neurorehabilitation for achieving rehabilitation treatment goals.

Regardless of the chosen treatment, there are 15 principles underlying effective neurorehabilitation that promotes neuroplasticity (Figure 9). (Maier et al., 2019) These principles govern the typology and timing of rehabilitative practice. Each temporal characteristic of the sessions and the duration of therapy must be planned to obtain the best physical and cognitive performance of the patient. Repetition during sessions and rest between repetitions improve the subject’s skills and induce profound changes in the brain’s ability to retain and process information (Anaya and Branscheidt, 2019). When planning a session, the specificity of the tasks must be considered, as this shapes the internal sensorimotor representation of the skill and facilitates motor learning and subsequent retention. When the session is goal-oriented, it promotes the achievement of a goal and leads to greater performance in motor learning than non-specific sessions (Afridi et al., 2023). Including variations of exercises in each rehabilitation session promotes generalization to similar tasks, even if these are not trained. Variability is correlated with greater neuronal activity and connectivity in the areas of the motor learning network during acquisition and is associated with better retention. If increasing difficulty is established throughout the treatment, it will result in a performance with fewer errors and less need to process information (Reid et al., 2016). During therapy, the subject’s exposure to multisensory information improves the ability to detect, discriminate, and recognise information, favouring connectivity between the motor and sensory cortices (Yang et al., 2021). The role of the therapist during the sessions is very important for sequencing the exercises and providing feedback to the patient. Rhythmic cues, usually auditory, facilitate motor execution and create a mental representation of the rhythm. The detection of regularity and the tracking of tempo increase activity in the areas of the motor network and the cerebellum (Nahum et al., 2013). Explicit feedback provides the subject with information about the quantitative or qualitative results of the task, correction, accuracy, success or failure, once the task is completed. Negative results can accelerate motor learning, while positive results ensure long-term retention. Implicit feedback is that which is given on the execution of the movement in the form of verbal descriptions, demonstrations or repetitions of recordings and which generates lasting adaptations (Silva-Batista et al., 2023). The therapist must also modulate the selection of effectors because not using a damaged muscle due to fear or comfort leads to a loss of neural function and correct behavior. Observing the action by the patient, as well as performing it, primarily recruits the premotor and parietal cerebral cortical areas, which could facilitate the execution of movement and motor learning by enhancing the excitability of the motor system. Performing mental practice gives the subject the ability to mentally simulate actions without overt behavior. This practice would recruit premotor areas, somatosensory cortex and subcortical areas as does effective practice, but also activation was found in the anterior cingulate cortex, which is linked to the cognitive aspects of motor control. The performance of group sessions benefits social interaction and the actions of one participant are both a response and a stimulus for the behavior of another. Self-perception of one’s own performance influences motor skill performance and learning but is also influenced by the evaluation or discouragement from others (Maier et al., 2019). Most patients with neurological damage will experience deficits that will change their lives beyond the acute phase. Therefore, neurorehabilitation is an important component of recovery. Although there are significant variations in the type of therapy, its duration and/or intensity; the application of the 15 principles based on neuroplasticity will facilitate the planning of neurorehabilitation treatment to achieve the established therapeutic goals.

## 6 Prevention

The consequences of dysphagia include malnutrition, dehydration, and in many cases, death from aspiration pneumonia (Mandell and Niederman, 2019). Prevention is presented as a tool to mitigate the deficits caused by impaired swallowing in older adults. Currently, there are no records of structured prevention practices because clinical practice shows that, often, both doctors and patients interpret dysphagia as a normal consequence of ageing and underestimate it (Tulunay-Ugur and Eibling, 2018).

Dysphagia prevention would only be possible before or during presbyphagia as the theory suggests that presbyphagia and dysphagia are on a continuum and that one progresses through this continuum as one ages (Namasivayam-MacDonald et al., 2018).Detection and assessment to determine swallowing disorders should be carried out as soon as possible in at-risk groups. Likewise, the adult population should be educated to self-detect early signs of dysphagia. The administration of subjective questionnaires, objective screening tests, dental and oral healthcare, attention to diet, and strength and endurance exercises for the swallowing muscles would, taken together, be an effective programme for the prevention of dysphagia during ageing (Chen et al., 2021).

The administration of a self-perception questionnaire to apparently healthy people over the age of 65 can enable the early diagnosis of presbyphagia and facilitate its rehabilitation (Cavallaro et al., 2022). The validated screening protocol for use with older adults living in the community and their caregivers and/or family members is the EAT-10 (Bartlett et al., 2022). Once self-perceived swallowing problems have been identified and to objectify the swallowing disorder, it is necessary to perform screening tests such as the GUSS, the V-VST, and also a test to assess chewing ability, the Test of Masticating and Swallowing Solids (TOMASS) (Huckabee et al., 2018) proposed for older adult patients who have well-fitted dental pieces and/or dentures. The results of objective screening tests will also facilitate the control of bolus volume and viscosity in the oral preparatory and oral propulsion stages for the ingestion of solids and liquids (Jiang et al., 2024). Dental care (replacement of missing teeth, repair of diseased teeth, and/or adjustment of dentures) improves chewing, bolus formation, and management. In turn, promoting oral hygiene will serve to reduce bacterial colonisation and prevent respiratory infections in case of penetration or aspiration. To complete the prevention plan, a strength and resistance exercise programme would be necessary (Carnaby et al., 2020). Although very little research has been done to elucidate the role of preventive exercises in the population, significant improvements in swallowing physiology have been found after the application of 8-week comprehensive swallowing treatment protocols (Balou et al., 2019) and tongue strengthening exercise programmes in older adults. These interventions, although scarce, have managed to increase isometric pressure and improve swallowing in older adults (Namasivayam-MacDonald et al., 2018).

## 7 New technologies

New technologies are a reality in our daily lives and have become tools that facilitate processes, both at home and in the workplace and healthcare (Sejdic et al., 2020). Artificial intelligence (AI) is a commonly used term to refer to the field of science aimed at equipping machines with the ability to perform functions such as logic, reasoning, planning, learning, and perception. Thus, these autonomous entities are capable of correctly interpreting external data, learning from that data, and using that knowledge to achieve specific tasks and goals through flexible adaptation (Kaplan and Haenlein, 2019). AI encompasses various technological aspects: planning, robotics, natural language processing, perception, knowledge, and machine learning. In medicine, machine learning has been used to create models for detecting and diagnosing diseases (Foster et al., 2014), for triage (Wu et al., 2020), for epidemiological analysis (Schneeweiss and Avorn, 2005), and as a support for clinical decision-making by predicting disease risk (Gultepe et al., 2014; Myszczynska et al., 2020) The usefulness of AI in dysphagia ranges from screening and detecting the disorder, even in asymptomatic patients, to the administration of rehabilitation treatments to patients who would otherwise not have had access to treatment (Coyle and Sejdić, 2020). Dysphagia screening in large populations is costly. For this purpose, an expert system (ES) based on machine learning has been developed, which calculates the risk of OD from the analysis of the clinical history data of all elderly hospitalized patients during their admission to a hospital. The software developed (AIMS-OD) provides systematic and universal screening of OD in real time during hospital admission, allowing the selection of the most appropriate diagnostic and therapeutic strategies for each patient (Martin et al., 2023). In the evaluation of dysphagia, the use of cervical auscultation (CA) has been implemented to analyse the sounds emanating from the neck during swallowing and which could reflect abnormal physiological events during the same (Borr et al., 2007). This evaluation method depends on the therapist who must be trained and have extensive experience. Currently, AI allows high-resolution cervical auscultation (HRCA) recordings to be made, through accelerometers placed on the patient’s neck. These recordings are analysed with a neural network trained to detect abnormal opening and closing of the UES, with over 90% accuracy (Coyle and Sejdić, 2020). In the laboratory, an adaptive reinforcement machine learning algorithm has been shown to have diagnostic value and efficacy for dysphagia by also analysing the patient’s swallowing sounds, recorded with an electronic stethoscope (Suzuki et al., 2022). The possibility of collecting data non-invasively through swallowing sounds has expanded the modalities of dysphagia assessment. The development of a real-time portable assessment tool, incorporated into a smartphone, allows for bedside patient assessment or remote assessment of those patients who are unable to move. This portable model (GOKURI) consists of three units: a neck band with a microphone, an amplifier, and an interface that displays results and statistics (Kou et al., 2022). Currently, the identification of a wet voice as a sign of aspiration depends on the experience and auditory ability of the evaluator. With new technologies, the spectrogram and voice data of a patient, performing four phonatory tasks, can be analysed with a deep learning model. These data, which are non-invasive to obtain, are a reliable tool for the diagnosis of dysphagia (Kim et al., 2023). Monitoring the displacement of the hyoid bone during swallowing in real time is another diagnostic method currently performed with VFS. In cases where the hyoid bone is lower and has little mobility, it suggests that the risk of dysphagia would be high. With machine learning and AI, it has been possible to avoid the radiation of VFS since the tracking of hyoid movement is performed with accelerometers placed on the patient’s neck and the data is analysed automatically. This method has an accuracy of over 89% (Matsuda et al., 2022). The use of new technologies has also extended to the treatment of dysphagia with the development of a prototype of an intelligent assistant that monitors adherence and provides feedback to the patient. This prototype is based on the need for the affected subject to visualise the practice, in order to promote neuroplasticity and self-perception. It is a mobile application that, when placed in front of the patient, acts as a mirror and a source of feedback to help with the use of safe compensatory strategies (Freed et al., 2016).

The integration of advanced technologies into the field of dysphagia management has significantly enhanced the diagnostic process and treatment outcomes. These new technologies offer more precise and objective assessments, reducing reliance on subjective evaluations. Early detection of dysphagia enables proactive intervention, mitigating the risk of complications such as aspiration pneumonia. Additionally, personalized treatment plans tailored to the individual characteristics and needs of the patient can optimize outcomes and improve patient satisfaction. Furthermore, remote monitoring and telemedicine capabilities expand access to dysphagia services, particularly in underserved regions. By streamlining diagnosis and treatment, these technologies can also lead to reduced healthcare expenditures.

While these advancements offer substantial benefits, the implementation of new technologies in dysphagia care is not free of challenges. Key areas for future research and development include ensuring the reliability and efficacy of these technologies through rigorous clinical validation. Moreover, integrating these technologies seamlessly into routine clinical practice is crucial to maximize their impact. Developing more sophisticated algorithms can further improve the accuracy and precision of diagnostic and treatment tools. Additionally, understanding the factors that influence the adoption and use of new technologies by patients and healthcare providers is essential for successful implementation. By addressing these limitations and investing in ongoing research, the full potential of new technologies in dysphagia management can be realized, leading to improved patient outcomes and a better quality of life.

## 8 Conclusion

Swallowing is a complex mechanism that involves cortical areas, five cranial nerves, the first three cervical segments, and muscles of the mouth, pharynx, and esophagus, coordinated from the brainstem. Dysphagia is a swallowing disorder that affects the quality of life of patients who suffer from it and endangers their lives due to the severity of its consequences. Presbyphagia, as a manifestation of physiological ageing, has been a commonly overlooked health problem, so it would be necessary to implement early detection and specific intervention measures. New technologies and AI generate possibilities for large-scale screening of dysphagia, prevention, and remote treatments implemented by assistive robotic platforms. The development of systems capable of interacting reliably with people through speech recognition, facial recognition, and real-time monitoring of functions and vital signs enables the early detection of signs. The prevention of dysphagia in ageing is possible, but it requires a multidisciplinary approach that combines early detection, adequate assessment, specific care programes, and new technologies, so that eating, during ageing, becomes satisfying and safe again.
